# Comparing the Severity of Injury and Trauma Pattern between Scooter and Street Motorcycle Riders; a Prospective Cohort Study 

**DOI:** 10.22037/aaem.v9i1.1229

**Published:** 2021-06-08

**Authors:** Mansour Bahardoust, Arman Karimi Behnagh, Abolfazl Bagherifard, Mehrdad Khodabandeh, Seyed Ali Emami, Shakiba Ghasemi Assl, Farid Najd Mazhar

**Affiliations:** 1 Bone and Joint Reconstruction Research Center, Shafa Orthopedic Hospital, Iran University of Medical Sciences, Tehran, Iran.; 2 Faculty of Medicine, Iran University of Medical Sciences, Tehran, Iran; 3 Neuromusculoskeletal Research Center, Department of Physical Medicine and Rehabilitation, Iran University of Medical Sciences

**Keywords:** Wounds and Injuries, Emergency Treatment, Accidents, Traffic, Motorcycles, Mortality

## Abstract

**Introduction:**

The popularity of motorcycle riding in Iran is increasing. However, there is a lack of information about the safety of different motorcycle types. This study aimed to compare the severity of injury and trauma pattern between scooter (vespa) and street (standard) motorcycle riders.

**Method:**

In a prospective cohort study, a comparison of demographics, injury severity, trauma pattern, and clinical characteristics between 324 riders (162 Vespa and 162 standard motorcycles) admitted to emergency departments was undertaken. The risk factors associated with severe injuries in the two groups were also determined. An emergency medicine specialist determined the severity of trauma based on the abbreviated injury scale (AIS).

**Results:**

The Odds Ratio (OR) of severe injuries was significantly higher in the standard motorcycle riders’ group (OR: 3.09; 95% CI: 1.9-4.21; p: 0.013). The frequency of lower extremity fractures was significantly lower in the Vespa group (OR: 4.11; 95% CI: 2.01-6.25; p = 0.012). The frequency of admission to the intensive care unit was significantly higher in the standard motorbike riders’ group (OR: 1.64; 95% CI: 1.11-2.51; p = 0.033). The multivariate analysis indicated that motorcycle type, the speed at the time of the accident, use of helmet, and age of riders are the most important predictors of trauma severity in riders (p<0.05).

**Conclusion:**

The pattern of injury varies between standard and Vespa motorcycles. The standard motorcycle riders were prone to a higher risk of adverse outcomes such as severe injuries. Due to the particular structure of scooters, the rate of lower limb injuries was significantly lower than standard motorcycles.

## 1. Introduction

Trauma is regarded as one of the major causes of death in developed countries, ranking high on the list of mortality and morbidity causes in younger populations. Traffic injuries are the leading cause of death in children and young adults (ages 5-29). Although ranked as the sixth major global mortality cause, it is predicted that fatality of traffic injuries will increase and it will rise to the fourth spot (1).  More than half of all road traffic deaths occur in vulnerable road users: pedestrians, cyclists, and motorcyclists (2). Injuries sustained in two-wheeled vehicle accidents are often more severe than those sustained in automobile accidents (3). Besides, globally, the probability of motorcycle riders being injured is about three times more than car occupants and they are sixteen times more probable to die due to road traffic injuries (4).

Low- and middle-income countries are disproportionately more burdened by the significant mortality and morbidity inflicted by motor-vehicular accidents (MVA) (5, 6). The most common causes of motorbike-related MVA's are illegal overtaking, excessive speeding, alcohol intoxication and substance abuse, motorbike type and safety, and not wearing standard protective gear such as helmets (7, 8).  In Iran, motorbikes are widely used, resulting in an increased frequency of motorbike-related MVAs that impose a high burden of disease on the healthcare system (5, 6, 9).

The MVA-associated mortality in motorcyclists, mainly occurs due to head traumas and helmets can reduce the regarded rate (10). Moreover, musculoskeletal injuries are common among motorized two-wheeled vehicle riders, and they are frequently associated with head and neck injuries. The lower extremities are the most common sites of orthopaedic injuries in a motorcycle collision. The incidence in some studies ranges between 40% and 60%, and limb entrapment is regarded as the most common mechanism of injury (11). 

**Figure 1:**
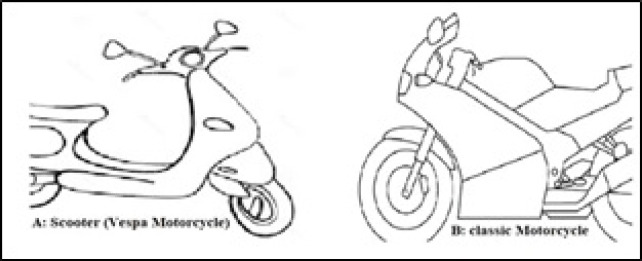
Scooter (Vespa) versus standard (Street) type of motorcycles

The range and variety of motorcycle models continue to grow. However, in this study, we focused on two types, standard and common street-legal motorcycle types and Vespa scooter (Figure 1). Scooters are primarily designed for use at low and medium speeds on urban streets. Relatively small in size with small-diameter wheels, their step-through design and general appearance differ significantly from full-sized motorcycles (12). Vespa scooters are designed with a particular guard for the legs. Moreover, compared to standard motorcycles, Vespa scooters, have lower speed, smaller engine capacities, and also smaller wheel with high manoeuvre capability, which provide an advantage of better safety profile. The number of studies tackling the differences in MVA-related trauma based on motorbike type is somewhat limited. However, few reports have documented less severe traumas in Vespa motorbikes compared to others. The current literature is mainly focused on the fatality of different motorcycle types and the coverage of other aspect of motorcycle-related collisions is limited. This prospective study aimed to compare the fatality and orthopaedic injury rates between Vespa motorbikes and other conventional street-legal motorbikes.

## 2. Methods

***2.1. Study Settings and design***

This prospective cohort study enrolled 324 motorbike riders involved in an intra-city motor-vehicular accident resulting in upper and lower limb fracture(s) admitted to the emergency departments (EDs) of hospitals affiliated to Iran University of Medical Sciences, Tehran, Iran, between May 2019 and May 2020. After assessing eligibility criteria, the patients were divided into two groups, based on the type of motorbike involved: the first group (n=162) comprised of Vespa-riders, while the second group (n=162) were riding standard motorbikes. To gain access to their medical records, patients were asked to fill out a written informed consent form. The study protocol was approved by Ethics committee of Iran University of Medical Sciences (Ethics code: IR.IUMS.REC.1398.288).

***2.2.*** ***Study Population***

Patients were enrolled through cluster sampling of EDs and during the study recruitment period all the patients with following criteria were recruited for this study: 1) involvement in an MVA while riding a Vespa or standard motorbike; 2) admission to ED; 3) availability of complete accident reports; 4) availability of complete motorbike documentation; 5) age between 18-50 years. Our exclusion criteria were as follows: 1) incomplete accident report; 2) occurrence of an incident out of the city bounds; 3) admission to EDs for reasons other than MVA (i.e., falling). The patients were matched for age, intoxication, speed, and engine power to control confounding factors.

***2.3. Data Source and Measurements***

Demographic data, age, gender, type of motorbike (scooter or standard), the speed at the time of the incident, intoxication with alcohol or other substances, mechanism of trauma, and status of using safety equipment were gathered using a predesigned checklist. Besides clinical and radiologic findings, including fracture location and severity, duration of hospitalization, intensive care unit admission, the cause of death, place of death, and the interval between accident and death were gathered using patients’ records and if possible, a brief interview was done (if they were alive). Fracture type and treatment procedures were classified based on the ICD-10 manual. The trauma mechanism was classified into three groups: driver error, vehicular factors, and environmental causes.

The details on the accident-associated parameters, such as type of motorbike, mechanism of accident, and speed of each motorbike at the time of accident were provided by exploring the police reports about the accident scene. An emergency medicine specialist determined the severity of trauma based on the abbreviated injury scale (AIS) (13). AIS evaluates trauma severity based on the extent of anatomical injuries. In this scaling system, various injuries are exclusively coded, and the overall severity is classified into six groups: mild, moderate, serious, severe, critical, and life-threatening. The data for each group was gathered separately. An orthopaedic surgeon determined the definitive location of the fracture(s) by examining radiologic images (14). 

***2.4. Statistical Analysis***

All the statistical analyses were performed using SPSS software for Windows v22. Descriptive analysis was performed and results were reported as mean and median for quantitative data and frequency and regarded percentage for qualitative data in both groups. The Kolmogorov Smirnov test was used to assess data normality. The t-test and non-parametric Mann Whitney U tests were used to compare normally distributed and otherwise data. The Chi-square test was used to compare quantitative data between the groups. Significance was reported using odds ratios (OR) and a 95% confidence interval (CI). Logistic regression was performed to determine the most important risk factors of the severity of trauma. The P-values <0.05 were deemed statistically significant. 

## 3. Results

***3.1. Baseline characteristics of studied cases***

This study enrolled a total of 324 motorbike riders (162 scooters and 162 standard motorcycles). The mean age of the study population was 28.01 ± 1.22 years (100% male). Table 1 compares the baseline characteristics of the study subjects between scooter and standard riders. The mean age for the Vespa and the traditional motorbike group were 27.90 ± 20.30 and 29.03 ± 21.22 years, respectively (p = 0.42). 104 (64.2%) standard and 98 (60.5%) scooters motorbike riders were exceeding 60km/h. No significant differences were observed between the two groups regarding age (p = 0.42), accident speed (p =0.11), helmet use (p = 0.079), the main cause of death (p = 0.28), place of death (p = 0.11), or time of death (p = 0.083). Severity of trauma based on AIS score was greater in the standard motorbike group (p = 0.001). 

***3.2. Comparing the location of injury and clinical outcomes***

Table 2 compares the location of injury and clinical outcomes between the two groups. The frequency of lower extremity fractures was significantly lower in the scooter group (OR: 4.11; CI 95%: 2.01-6.25; p = 0.012). In the standard motorbike group, the most common fracture locations were the knee, the shin, the ankle, and the foot. However, the frequency of these fractures was significantly lower in the scooter group (P=0.001). The two groups were similar regarding trauma to the head and neck (p > 0.081) and other limbs (p > 0.05). The overall mortality (p=0.084) and duration of hospital stay (p=0.76) were similar in the two groups. The frequency of admission to the intensive care unit was significantly higher in the standard motorbike riders’ group (17.9% versus 9.9%; p = 0.033).

***3.3. Risk factors of severe and critical trauma***

In general, the trauma was severe or critical in 100 (30.9%) of riders, based on the AIS. We used adjusted logistic regression analysis to control confounders in evaluation of factors associated with severe trauma. All variables with statistical significance in univariate analysis were included in Logistic regression. The multivariate analysis indicated that age < 24 years (Odds: 2.53; 95%CI: 1.46 – 3.66; p = 0.012), accident speed ≥ 60 km/hours (Odds: 3.56; 95%CI: 2.12 – 5.10; p = 0.001), use of a helmet (Odds: 0.66; 95%CI: 0.46 – 0.87; p = 0.001), and standard motorcycle type (Odds: 1.47; 95%CI: 1.07 – 1.98; p = 0.018) were among the most important risk factors of trauma severity in this study.

## 4. Discussion

This study characterized subnational data on patients admitted to the ED who were involved in standard street-based motorcycles or Vespa scooters. Our data suggest that Vespa scooters possess better safety profile in comparison to motorcycles. Although the two groups did not show any difference in terms of mortality rate and duration of hospital stay, the number patients admitted to ICU was significantly higher in motorcycle riders, which implies much severe injuries among the motorcyclists. Moreover, site analysis of the injuries showed that motorcycle riders experienced a higher frequency of injuries in lower extremities. Furthermore, analysis of the factors associated with severity of injuries, revealed that type of vehicle was one of the factors that could affect the severity of the injuries and the riding of standard street-based motorcycles can lead to more severe injuries. 

The prevalence of using low-speed motorcycles such as scooters has been increasing. This mainly owes to the affordability and low fuel consumption of this type of vehicle (15). Previous reports on the safety profile of scooters revealed that this increase in riding this type of vehicle may lead to a higher burden of disease (15, 16). However, these reports mainly focused on low-speed motorcycles. Although the higher consumption rate of this type of motorcycle can inflate the regarded burden of disease, studies similar to our work revealed that overall, low-speed motorcycles such as vespa scooters showed better safety profiles (17, 18). The use of either types of motorcycle can be associated with injuries including fractures and internal organ damages. However, the pattern and distribution of injuries showed discrepancy between the two groups. 

According to our analysis, vespa riders had a higher percentage of injuries to head/neck and upper extremities; however, these observations failed to have statistical significance. On the other hand, motorcycle riders showed considerably more injuries in lower extremities. Our findings are inconsistent with the different injury patterns previously described, with the upper extremity more common in motorcycle riders and injury to lower extremities were more common in scooter or moped riders (18, 19). The source of discrepancy can be that the type of scooter and also counting moped and scooter as one group would change the safety profile of the scooters reported in studies. Besides, head injuries, with a higher frequency among motorcycle riders, were considered the most common cause of death in both groups (18). Like upper extremity injuries, the percentage of injury to head and neck was higher in vespa group. An explanation for this observation was provided by White et al. Based on their study, scooter riders were less likely to use safety equipment (18). Also, the previous reports showed that using safety equipment such as helmets could reduce the risk of death and severe injuries (10). Furthermore, in an epidemiologic study in Iran, 23% of all fatal traffic injuries belonged to motorcycle riders, among whom 59% of all mortalities were due to head injuries. Most motorcycle fatalities belonged to the 18-24 years age group (29.1%), and also, the overall percentage of safety helmet use among motorcycle accident victims was estimated at 37.4% (9). In this study, the mortality rate was not different between the two study groups. However, riding a motorcycle can increase the risk of severe injuries. In a comparative study in Netherlands (19), it was demonstrated that the vehicle type being light moped was among the factors that increased the risk of severe injuries. 

Interestingly, the speed at the time of accident and death did not differ significantly between the two study groups. Although the number of cases who died on the accident scene was higher in the motorcycle group (64.2% vs. 60.5%), this difference was not significant. Nonetheless, this finding may imply that the severity of accidents and, consequently, the regarded injury at the time of the accident were considerably higher among motorcycle riders. In another survey by Blackman et al. (17), the factors influencing accident severity among motorcycle, moped, and scooter riders were evaluated. Our study and other reports showed that riding motorcycles were associated with a higher rate of severe accidents in comparison to scooters. Moreover, they reported that one reason for such observation could be the use of each vehicle (17). For instance, scooters are mainly used for short distances and usually at low speed. However, the motorcycle is primarily used in long distances and therefore, used at high speeds. Nonetheless, in their study and other surveys including the present study, speed did not play a pivotal role in the severity of injuries or mortality rates (10, 17, 18). 

 There was no significant difference between the two groups regarding high or low speed riding in our study, and the prevalence of above 60 km/h was similar in the two study groups. Also, we observed that most of the collisions occurred at the speed range of 50-75 km/h. Nevertheless, this can mislead us since this categorization did not indicate the prevalence of very high speed, i.e., above 90 km/h in each group. Inconsistent with our observation, Blackman et al. (17) showed that speed above 80 km/h is associated with a high risk of a severe accident in motorcycle riders. Similarly, the speed limits for mopeds and scooters were 90 km/h and 70 km/h, respectively.

There was another factor that affected the severity of the accidents regardless of the vehicle type. Younger age was associated with more severe accidents. However, this finding was inconsistent with others reports in the literature, indicating that the older ages, specially above 75 years, is associated with undesirable outcomes among scooter and motorcycle riders (17, 19-21). One possible explanation for this discrepancy can be the fact that all the mentioned studies were conducted in western countries. According to our literature search, in the middle eastern countries, younger age is associated with severe injuries among the motorcycle riders (22). In a comprehensive analysis of motorcycle pattern in Iran, it was demonstrated that the majority of motorbike injuries had happened in younger ages. It was demonstrated that the a great deal of young riders did not have any motorcycle riding license making them vulnerable to motorbike-associated injuries (23).

**Figure 2:**
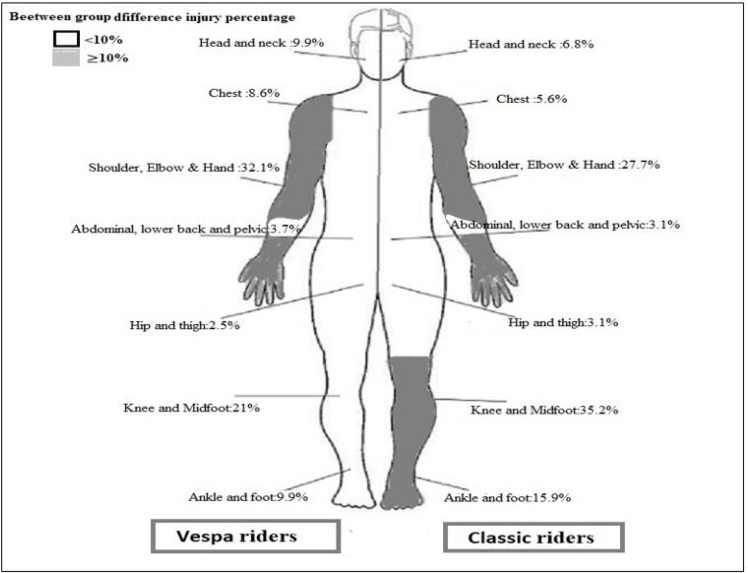
Prevalence of traumatic injuries in scooter (Vespa) and standard (Street) motorcycle riders

**Table 1:** Comparing the baseline characteristics of studied participants between scooter (Vespa) and standard (Street) motorcycle riders

**Variable**	**Motorcycle Type**		**P**
	**Standard (n:162)**	**Scooter (n:162)**	
**Age (Year) **			
Mean ± SD	29.03 ± 21.22	27.90 ± 20.30	0.56
16-24	69 (42.6)	76 (46.9)	0.42
25-39	59 (36.4)	58 (35.8)	
≥40	34 (21.0)	28 (17.3)	
**The speed at the time of the accident (Km/h)**			
<60	58 (35.8)	64 (39.5)	0.11
≥60	104 (64.2)	98 (60.5)	
**Helmet use**			
Yes	101 (62.4)	92 (56.7)	0.079
**Helmet use in dead riders **			
Yes	4/18 (22.2)	4/16 (25.0)	0.062
**The direct cause of death**			
Head and neck injury	13/18 (72.2)	12/16 (75.0)	0.28
Multiple injuries	5/18 (27.8)	4/16 (25.0)	
**Death place**			
On the scene of accident	11/18 (61.1)	8/16 (50.0)	0.11
On the way	2/18 (11.1)	2/16 (12.5)	
In a health facility	5/18 (27.8)	6/16 (37.5)	
**Time to death (hours)**			
**<** 24	12/18 (61.1)	10/16 (62.5)	0.83
≥ 24	6/18 (39.9)	6/16 (37.5)	
**Blood alcohol **			
Positive	11 (6.8)	9 (5.6)	0.89
**Addiction History **			
Yes	15 (9.3)	14 (8.5)	0.19
Trauma Severity*			
Minor	44 (27.2)	64 (39.5)	0.035
Moderate	50 (30.9)	66 (40.8)	0.08
Serious	40 (24.7)	18 (11.1)	0.013
Severe	28 (17.2)	14 (8.6)	0.001

Data are presented as mean ± standard deviation or frequency (%).*: based on AIS score

**Table 2:** Comparing fracture location and clinical outcomes between scooter (Vespa) and standard (Street) motorcycle riders

**Variable**	**Motorcycle type**		**P**
	**Standard (n:162)**	**Scooter (n:162)**	
**Fracture Location**			
None	7 (4.3)	20 (12.3)	0.001
Upper extremity	65 (40.1)	82 (50.7)	0.88
Lower extremity	90 (55.6)	60 (37)	0.012
Head and neck	11 (6.8)	16 (9.9)	0.081
Chest	9 (5.6)	14 (8.6)	0.14
Shoulder, Elbow & Hand	45 (27.7)	52 (32.1)	0.11
Abdominal, lower back, and pelvic	5 (3.1)	6 (3.7)	0.21
Hip and thigh	5 (3.1)	4 (2.5)	0.38
Knee and Midfoot	57 (35.2)	34 (21 )	0.001
Ankle and foot	16 (15.9)	16 (9.9)	0.018
Minor damage	5 (3.1)	20 (12.3)	0.001
**Outcomes**			
Death	18 (11.1)	16 (9.9)	0.084
Hospital stay (day)	6.6 ± 4.9	5.1±3.8	0.076
Admission to ICU	29 (17.9)	16 (9.9)	0.033

Data are presented as mean ± standard deviation or frequency (%). ICU: intensive care unit.

**Table 3:** Multivariate logistic regression analysis of factors associated with severe and critical trauma in motorcycle riders

**P**	**95% CI**	**Odd**	**Variable**
0.012	1.46-3.66	2.56	**Age < 24 years**
0.001	2.12-5.10	3.56	**Speed ≥ 60 (Km/h)**
0.001	0.46-0.87	0.66	**Use of helmet**
0.018	1.07-1.98	1.47	**Motorcycle (Standard type)**

Odd: Adjusted odds ratio, CI: confidence interval.

## 5. Strengths and limitations

Our study had some strengths and weak points. The main strong point of this study was a considerably large number of included participants in each group. Besides, this study was designed using a prospective methodology. Moreover, unlike the previous reports, the rate of pre-hospital deaths was determined in this study. Also, the effect of some confounders such as alcohol intoxication was adjusted in this study, which had not been achieved in studies pursuing similar objectives. The study's main weakness was that the extraction of several variables such as the mechanism of the accident from police reports limited the classification of injuries based on accident mechanism. Also, we failed to follow the patients to understand the post-ED outcomes of the study participants, which could provide insight on the burden of disease provided by each motorcycle type. 

## 6. Conclusion

This study suggests that riders of Vespa scooters and motorcycles may have different patterns of injury. The motorcycles possess a higher risk of severe outcomes, such as higher fatality and severe injuries in comparison to Vespa scooters. Further evaluation of the injuries sustained by Vespa riders is required to understand their impact more fully.

## 7. Declarations

### 7.1. Data availability statement

The datasets used and analyzed during the current study are available via the corresponding author on reasonable request.

### 7.2. Acknowledgments

Hereby, the authors would like to express gratitude to the Vice-Chancellor for research, Iran University of Medical Sciences. 

### 7.3. Funding

The authors would like to express gratitude to the Vice-Chancellor for research, Iran University of Medical Sciences.

### 7.4. Conflict of interest

The authors have no conflicts of interest to declare for this study

### 7.5. Author contributions

All authors meet the standard criteria of authorship contribution based on the recommendations of the International Committee of Medical Journal Editor. All authors listed on the title page have read and approved the manuscript, attest to the validity and legitimacy of the data and its interpretation, and agree to its submission to " Archives of Academic Emergency Medicine" for evaluation and review for possible publishing. 

### 7.6. Ethics approval

This study was reviewed and approved by the Ethics Committee of Iran University of Medical Sciences (IR.IUMS.REC.1398.288), and informed consent was obtained from participants before their inclusion in the study.
